# Label-Efficient PCB Defect Detection with an ECA–DCN-Lite–BiFPN–CARAFE-Enhanced YOLOv5 and Single-Stage Semi-Supervision

**DOI:** 10.3390/s25237283

**Published:** 2025-11-29

**Authors:** Zhenxia Wang, Nurulazlina Ramli, Tzer Hwai Gilbert Thio

**Affiliations:** 1 Centre for Sustainability in Advanced Electrical and Electronics Systems (CSAEES), Faculty of Engineering, Built Environment and Information Technology, SEGi University, Petaling Jaya 47810, Malaysia; 2 Department of Electrical Engineering, Hebei Vocational University of Technology and Engineering, Xingtai 054000, China

**Keywords:** PCB defect detection, semi-supervised object detection, YOLO, Innovation and Infrastructure

## Abstract

Printed circuit board (PCB) defect detection is critical to manufacturing quality, yet tiny, low-contrast defects and limited annotations challenge conventional systems. This study develops an ECA–DCN-lite–BiFPN–CARAFE-enhanced YOLOv5 detector by modifying You Only Look Once (YOLO) version 5 (YOLOv5) with Efficient Channel Attention (ECA) for channel re-weighting, a lightweight Deformable Convolution (DCN-lite) for geometric adaptability, a Bi-Directional Feature Pyramid Network (BiFPN) for multi-scale fusion, and Content-Aware ReAssembly of FEatures (CARAFE) for content-aware upsampling. A single-cycle semi-supervised training pipeline is further introduced: a detector trained on labeled images generates high-confidence pseudo-labels for unlabeled data, and the combined set is used for retraining without ratio heuristics. Evaluated on PKU-PCB under label-scarce regimes, the full model improves supervised mean Average Precision at an Intersection-over-Union threshold of 0.5 (mAP@0.5) from 0.870 (baseline) to 0.910, and reaches 0.943 mAP@0.5 with semi-supervision, with consistent class-wise gains and faster convergence. Ablation experiments validate the contribution of each module and identify robust pseudo-label thresholds, while comparisons with recent YOLO variants show favorable accuracy–efficiency trade-offs. These findings indicate that the proposed design delivers accurate, label-efficient PCB inspection suitable for Automated Optical Inspection (AOI) in production environments. This work supports SDG 9 by enhancing intelligent manufacturing systems through reliable, high-precision AI-driven PCB inspection.

## 1. Introduction

Printed circuit boards (PCBs) form the backbone of electronic systems by mechanically supporting and electrically connecting components. Even minor PCB surface defects—such as scratches, open circuits, or solder shorts—can cause malfunctions and degrade overall system performance [1]. Ensuring product quality and reliability therefore requires effective PCB defect detection. Traditionally, manufacturers have relied on manual visual inspection and automated optical inspection (AOI) for PCB quality control [2]. Manual inspection is labor-intensive and prone to inconsistency, while conventional machine-vision methods struggle with the complexity of PCB patterns and the diverse appearances of defects [2]. AOI techniques (e.g., template comparison or design-rule checking) can detect many defects but often require strict image alignment and controlled lighting; they also have difficulty generalizing to new defect types [3]. In practice, as new defect patterns continually emerge with changes in manufacturing processes, rule-based AOI systems must be frequently recalibrated to handle unseen anomalies [3]. These limitations, combined with the subjective and error-prone nature of human inspection, have driven a shift towards deep learning-based approaches for PCB defect detection [4].

Deep learning, especially convolutional neural networks (CNNs), can automatically learn discriminative visual features and has achieved superior accuracy in general image recognition tasks—in some cases even approaching or exceeding human-level performance [5]. By leveraging CNN models, PCB inspection systems become more adaptable to diverse or subtle defects without requiring explicit modeling of each defect type. In recent years, numerous studies have applied deep CNNs to PCB defect detection and reported significant improvements in detection accuracy over traditional methods [4]. For example, a 2021 study by Kim et al. developed a skip-connected convolutional autoencoder to identify PCB defects and achieved a detection rate up to 98% with false alarm rate below 2% on a challenging dataset [1]. This demonstrates the potential of deep learning to provide both high sensitivity and reliability in detecting tiny flaws on PCB surfaces, which is critical for preventing failures in downstream electronics.

Object detection models based on deep learning now dominate state-of-the-art PCB inspection research [6]. In particular, one-stage detectors such as the You Only Look Once (YOLO) family have gained popularity for industrial defect detection due to their real-time speed and high accuracy [7,8]. Unlike two-stage detectors (e.g., Faster R-CNN) that first generate region proposals and then classify them, one-stage YOLO models directly predict bounding boxes and classes in a single forward pass—making them highly efficient [6,8]. Early works demonstrated the promise of YOLO for PCB defect detection. For example, Adibhatla et al. applied a 24-layer YOLO-based CNN to PCB images and achieved over 98% defect detection accuracy, outperforming earlier vision algorithms [8]. Subsequent studies have confirmed YOLO’s advantages in this domain, showing that modern YOLO variants can even rival or surpass two-stage methods in both detection precision and speed [6,8]. The YOLO series has evolved rapidly—from v1 through v8 and, most recently, up to v11—with progressive architectural and training refinements (e.g., stronger backbones, decoupled/anchor-free heads, improved multi-scale fusion, advanced data augmentation, and Intersection-over-Union (IoU) aware losses) that collectively enhance accuracy–latency trade-offs across application domains [9]. For instance, the latest YOLO models employ features like cross-stage partial networks, mosaic data augmentation, and CIoU/DIoU losses to better detect small objects and improve localization [10,11]. YOLOv5, in particular, has become a widely adopted baseline in PCB defect inspection, valued for its strong balance of accuracy and efficiency in finding tiny, low-contrast flaws in high-resolution PCB images [12,13]. Open-source implementations of YOLOv5 provide multiple model sizes (e.g., YOLOv5s, m, l, x) that can be chosen to trade off speed and accuracy, facilitating deployment in real-world production settings [12]. However, standard YOLO models still encounter difficulties with certain PCB inspection challenges, such as extremely small defect targets, complex background noise, and limited training data. This has motivated researchers to embed additional modules into the YOLO framework and to explore semi-supervised training strategies tailored to PCB defect detection.

Beyond CNN-based detectors, Transformer-based architectures have recently emerged as another powerful paradigm for object detection [14,15]. Detection Transformer (DETR) and its successors formulate detection as a set prediction problem with a Transformer encoder–decoder, removing hand-crafted components such as anchors and non-maximum suppression while achieving competitive accuracy on Common Objects in Context(COCO) [16]. Vision Transformers such as Swin Transformer have also been adopted as general-purpose backbones for detection and segmentation, providing strong multi-scale features via shifted window self-attention [17]. Motivated by these advances, several works have begun to explore Transformer-based models for PCB defect inspection, including Transformer–YOLO hybrids [18,19] and real-time detection Transformers tailored to bare PCB inspection (e.g., Lite-DETR, Hierarchical Scale-Aware Real-Time Detection Transformer (HSA-RTDETR), and Multi-Residual Coupled Detection Transformer (MRC-DETR)) [20,21,22]. These methods demonstrate that global self-attention and set-based decoding can further improve defect detection, but they typically rely on large-scale pre-training, longer training schedules, and heavier computation, which may complicate deployment in resource-constrained AOI systems [18,20].

One major challenge in PCB defect inspection is the very small size and subtle appearance of many defect types (e.g., pinhole voids, hairline copper breaks). These tiny defects may occupy only a few pixels and can be easily missed against intricate PCB background patterns [4]. To address this, recent works have integrated attention mechanisms into YOLO detectors to help the network focus on important features. In particular, channel attention modules such as the Squeeze-and-Excitation (SE) and Convolutional Block Attention Module (CBAM) have been added to emphasize defect-relevant feature channels and suppress irrelevant background information [23]. For example, Xu et al. reported that inserting a CBAM module into a YOLOv5-based model improved recognition of intricate, small PCB defects under complex backgrounds by enhancing the model’s attention to critical regions [24]. A lightweight variant, Efficient Channel Attention (ECA), has proved effective in detection settings; by applying a short 1-D convolution to model local cross-channel dependencies—without the dimensionality reduction used in SE/CBAM—ECA enhances feature saliency with negligible computational overhead [25]. Kim et al. demonstrated that adding an ECA module into a YOLOv5 backbone boosted the detection of small objects in aerial images, as the channel attention helped highlight faint targets against cluttered backgrounds [25]. Similarly, an enhanced YOLOv5 model for surface inspection found that integrating ECA improved the identification of fine defects (especially tiny or low-contrast features) compared to using SE attention alone [26]. These findings underscore that incorporating efficient attention mechanisms enables YOLO models to better capture subtle defect cues that might otherwise be overlooked. A streamlined ECA module is embedded in the YOLOv5 backbone to adaptively accentuate faint PCB defect patterns, enabling clearer separation of true defect signals from background circuitry.

Another limitation of vanilla YOLO detectors lies in the fixed sampling grid of standard convolutions, which restricts the receptive field from conforming to irregular defect geometries on PCB. Deformable Convolutional Networks (DCN) alleviate this constraint by learning location-dependent offsets so that kernels adaptively sample informative positions, effectively “bending” to follow fine discontinuities, burrs, and spurious copper patterns. By aligning the sampling lattice with true object boundaries, deformable convolutions help prevent the mixing of faint defect signals with background textures and thereby preserve small-object detail during feature extraction [11]. Recent journal studies show that inserting a deformable layer into YOLO backbones or necks yields measurable gains on small-object benchmarks by retaining object cues and reducing background interference [27,28]. In the PCB context, improved YOLO variants that integrate DCN (or DCNv2) into high-resolution feature paths report enhanced localization of tiny, irregular defects and higher mean Average Precision, attributable to better spatial alignment around hairline breaks and micro-holes [29]. Beyond PCB imagery, complementary evidence from aerial and industrial surface datasets confirms that lightweight DCN blocks can be deployed with modest computational overhead to sharpen feature selectivity on thin, elongated structures—an effect particularly valuable for defect edges and gaps [28,30]. Following these insights, a DCN-lite layer is placed in the YOLOv5 neck to introduce spatial flexibility where fine spatial detail is most critical, aiming to increase sensitivity to minute or oddly shaped PCB anomalies while preserving throughput [29,31].

Effective multi-scale feature fusion is essential in PCB inspection, where target sizes span from large solder bridges to sub-pixel pinholes. While the original YOLOv5 neck adopts a Path Aggregation Network (PANet), recent work shows that bi-directional pyramid designs with learnable fusion weights strengthen small-object representations and improve robustness to scale variation. In particular, Bi-Directional Feature Pyramid Networks (BiFPN) iteratively propagate information top-down and bottom-up, balancing low-level spatial detail with high-level semantics and yielding consistent gains over PANet-style necks in one-stage detectors [32]. Journal studies report that replacing or augmenting PANet with BiFPN leads to higher precision and recall on small targets by avoiding attenuation of fine details during fusion [33]. In PCB-focused research, lightweight YOLO variants that integrate BiFPN in the neck achieve superior accuracy on micro-defects, indicating that normalized, weighted cross-scale aggregation is particularly beneficial for tiny, low-contrast structures [34]. Beyond PCB imagery, enhanced (augmented/weighted) BiFPN formulations further validate these trends in diverse vision tasks, demonstrating that learnable cross-scale weights can reduce information loss and emphasize discriminative cues at small scales [35]. Guided by this evidence, the proposed model adopts a BiFPN neck to more effectively blend high-resolution detail and contextual semantics before detection, improving sensitivity to both macro-level faults and minute solder splashes [36,37].

In addition to stronger feature fusion, refining the upsampling operator in the neck materially benefits small-defect detection. Fixed schemes (e.g., nearest-neighbor) can blur fine edges and attenuate weak responses, causing misses on hairline cracks or pinholes [38]. A learnable alternative is Content-Aware ReAssembly of Features(CARAFE), which predicts position-specific reassembly kernels from local content and reconstructs high-resolution features with a larger effective receptive field [39]. Unlike fixed interpolation, CARAFE preserves boundary and texture cues during upscaling and has been shown to improve one-stage detectors on cluttered scenes with numerous tiny targets [40]. Recent journal studies report that inserting CARAFE into YOLO-style necks yields higher precision/recall on small objects while maintaining real-time feasibility due to the module’s lightweight design [41]. Further evidence from remote-sensing benchmarks indicates that CARAFE reduces information loss and better aligns multi-scale features compared with naïve interpolation, boosting mAP for dense small targets [42]. Guided by these results, the present YOLOv5-based architecture replaces nearest-neighbor upsampling with CARAFE at top-down pathways to retain minute PCB defect details during feature magnification and to strengthen the downstream detector’s sensitivity to thin, low-contrast flaws [43].

While architectural enhancements increase capacity, data scarcity and class imbalance remain practical bottlenecks in PCB defect inspection. In early production or when new defect modes emerge, only a handful of labeled samples may exist, making fully supervised training prone to overfitting and poor generalization. Semi-supervised object detection (SSOD) addresses this by exploiting large pools of unlabeled imagery together with few labels, commonly through pseudo-labeling and consistency regularization in teacher–student schemes [44,45]. This setting aligns well with PCB lines, where acquiring images at scale is easy but fine-grained annotation is costly; leveraging unlabeled frames expands the distribution of backgrounds, lighting, and rare defects seen during training [44,46]. Recent journal studies demonstrate that filtering uncurated unlabeled sets and enforcing consistency across augmentations markedly improves pseudo-label quality and downstream detection, boosting mAP in low-label regimes [44]. Practical SSOD variants also integrate adaptive thresholds or active selection to suppress noisy pseudo-boxes while retaining diverse positives, further stabilizing one-stage detectors [47,48]. Guided by these findings, a single-cycle self-training pipeline is adopted: a detector trained on labeled PCB images generates high-confidence pseudo-labels on unlabeled data; the labeled and pseudo-labeled samples are then mixed without ratio heuristics for retraining, improving recall of subtle anomalies while keeping computational overhead modest [45,49]. In effect, training on both labeled and pseudo-labeled data broadens coverage of rare, small, and low-contrast defects, reducing false negatives and improving robustness in deployment [45,49].

However, existing PCB-oriented defect detectors still leave several practical gaps. Many YOLO-based variants assume fully annotated training sets and do not exploit the abundant unlabeled PCB images available on production lines. Other works focus solely on architectural modifications but do not systematically address the simultaneous requirements of (i) high sensitivity to tiny, low-contrast defects, (ii) operation under label-scarce regimes, and (iii) constrained computational budgets for deployment. As a result, there is still a lack of a deployment-ready, label-efficient one-stage PCB detector that jointly enhances small-defect representation and leverages unlabeled data.

Therefore, the objective of this study is to develop and evaluate a task-aligned, label-efficient PCB defect detector. We augment YOLOv5 with ECA, DCN-lite, BiFPN and CARAFE to strengthen multi-scale feature representation at modest computational cost. In the remainder of this paper, we refer to this architecture as the ECA–DCN-lite–BiFPN–CARAFE-enhanced YOLOv5 (the proposed model). In addition, we design a simple single-cycle semi-supervised training scheme that uses confidence-thresholded pseudo-labels on unlabeled PCB images to expand the effective training set. The effectiveness of the proposed model detector is then validated on the PKU-PCB dataset under different label-scarce regimes, with ablation studies and comparisons to recent YOLO variants. Rather than introducing entirely new backbone blocks, this work focuses on a task-aligned integration of existing attention, deformable, and feature-fusion modules and on a simple yet effective semi-supervised training scheme, with an emphasis on label efficiency and deployability for PCB AOI. Relevance to the Sustainable Development Goals. In the context of smart manufacturing, accurate and timely inspection is a cornerstone of resilient industrial infrastructure (SDG 9). By improving defect detection under label-scarce regimes, the proposed approach lowers the dependence on extensive manual annotation and supports scalable deployment of AI-enabled automated optical inspection.

## 2. Methods

**Figure 1:**
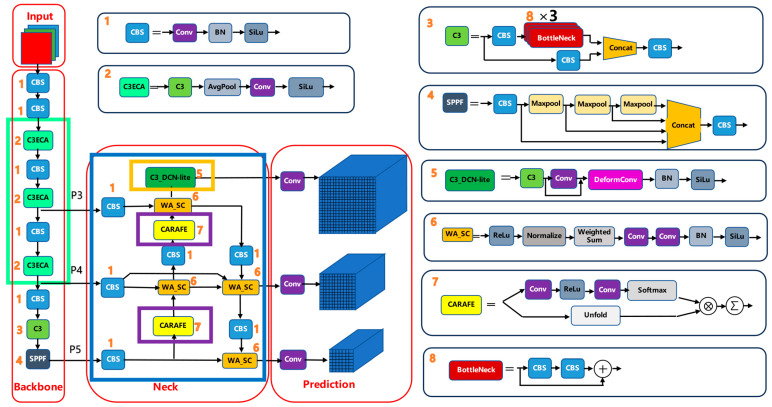
Overall architecture of the proposed ECA–DCN-lite–BiFPN–CARAFE-enhanced YOLOv5 model. Colored boxes highlight the newly added modules: green—ECA blocks in the C3 backbone stages; orange—the DCN-lite block on the P3 (stride-8) path; blue—BiFPN fusion nodes using the WA_SC block; purple—CARAFE upsampling operators in the top-down paths. Numbers shown next to the blocks are only reference indices without additional technical meaning.

**Figure 6:**
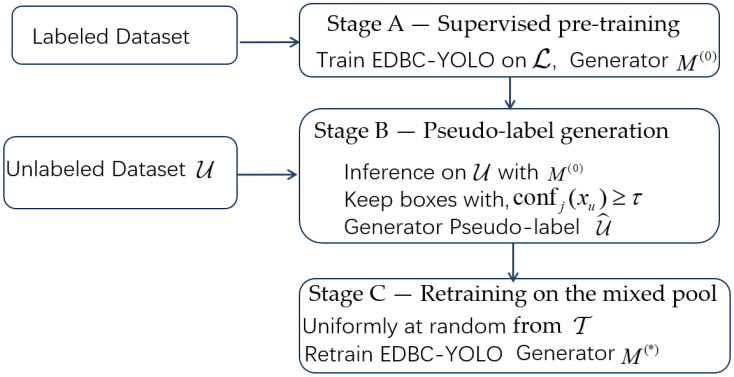
Flowchart of the single-cycle semi-supervised training pipeline.

Our approach comprises two main components: (1) a modified YOLOv5-based architecture with an enhanced backbone and neck (incorporating ECA, DCN-lite, BiFPN, and CARAFE modules), and (2) a one-stage semi-supervised training pipeline that leverages unlabeled data via pseudo-labeling. Readers who are mainly interested in the overall idea and results can focus on Figure 1 and Figure 6 together with the short summary in Section 2.3, and then proceed directly to Section 3. The following subsections provide more detailed descriptions of each module and the training procedure for readers who wish to reproduce or extend the method. Each component is detailed below.

### 2.1. Network Architecture: ECA–DCN-Lite–BiFPN–CARAFE-Enhanced YOLOv5

An overview of the modified YOLOv5 architecture is shown in Figure 1 above. The network is built on a YOLOv5 backbone with four key module enhancements aimed at improving feature extraction and detection of small defects:

ECA: Inserted by replacing the standard C3 blocks with C3ECA at the P2, P3 and P4 stages (strides 4, 8 and 16) of the YOLOv5 backbone to adaptively re-weight feature channels.

DCN-lite: A single lightweight deformable block (C3_DCNLite) is placed on the high-resolution P3 branch (stride 8) after the top-down BiFPN fusion at P3 and before the stride-8 detection head, so that deformable sampling focuses on the smallest defects.

BiFPN with WA_SC: The original PANet neck is replaced by a bidirectional BiFPN operating on P5, P4 and P3. Before fusion, P5 and P4 features are projected to 640 channels and P3 to 320 channels by 1 × 1 lateral convolutions, and each fusion node applies a WA_SC block (WeightedAdd + Separable Convolution).

CARAFE upsampling: In the top-down path (P5→P4 and P4→P3), CARAFE is used as the upsampling operator with factor 2, replacing fixed interpolation and preserving fine defect structures.

#### 2.1.1. Notation and Topology

Let the input image be $x\in {\mathbb{R}}^{3\times S\times S}$ (default S = 640). The backbone outputs feature maps $\left(P_{3},P_{4},P_{5}\right)$ at strides (8, 16, 32). Lateral 1×1 convolutions align channels before fusion: P5 and P4 are projected to 640 channels and P3 to 320 channels by 1 × 1 convolutions, as reflected in the final YOLOv5 configuration file (Appendix A). This ensures that all inputs to a given fusion node have the same channel dimension before applying WA_SC. The neck performs a top-down pass (P5→P4→P3) using CARAFE upsampling and a bottom-up pass (P3→P4→P5) using strided depthwise separable convolutions. Each fusion node applies a WeightedAdd operation to its inputs, followed by a depthwise separable refinement. The detector head predicts at strides 8, 16 and 32.

#### 2.1.2. ECA Inside C3

**Figure 2:**
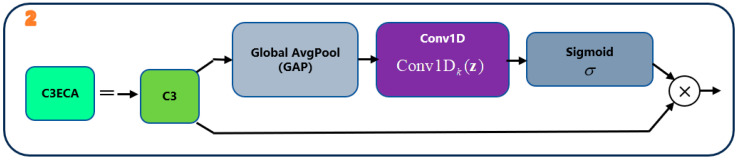
ECA block inserted into the C3 module. Global average pooling (GAP) aggregates each channel into a scalar, a small 1D convolution and sigmoid generate channel-wise weights, and these weights rescale the original feature map. The number ‘2’ indicates the C3ECA module.

Intuitively, the ECA module lets each channel look at a small neighborhood of channels and decide how important they are, using a tiny 1D convolution. Channels that are more informative for PCB defects receive higher weights, while less useful channels are suppressed, and this is done without adding a large number of extra parameters. As illustrated in Figure 2, the ECA block first applies global average pooling (GAP) to obtain a per-channel descriptor, then passes it through a small 1D convolution and a sigmoid to produce channel-wise weights, which are finally multiplied back to the original feature map. For a feature tensor $X\in {\mathbb{R}}^{C\times H\times W}$, global average pooling produces a per-channel descriptor:

(1) $$z_{c}=\mathrm{GAP}(X_{c})=\frac{1}{H\cdot W}\sum_{h=1}^{H}\sum_{w=1}^{W}X_{c}(h,w).$$

A small 1D convolution and a sigmoid activation then generate channel-wise weights:

(2) $$a=\sigma \left(\mathrm{Conv}1D_{k}(z)\right).$$

and the output feature map is

(3) $$Y_{c}(h,w)=a_{c}\cdot X_{c}(h,w).$$

The kernel size k of the 1D convolution is a small odd number determined by the channel count C, as in the original ECA design. ECA thus re-weights channels without dimensionality reduction, preserving efficiency. The internal computation of the ECA block inserted into C3 is illustrated in Figure 2.

#### 2.1.3. CARAFE Content-Aware Upsampling

**Figure 3:**
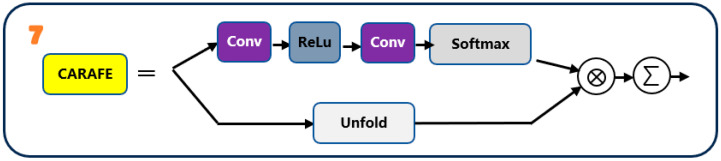
CARAFE content-aware upsampling in the neck. A content encoder compresses local neighborhoods, a kernel predictor generates position-specific reassembly kernels, and the reassembly operator uses these kernels to upsample the feature map. The number ‘7’ indicates the CARAFE upsampling module.

Following the CARAFE design, shown schematically in Figure 3, the module consists of a content encoder, a kernel prediction module and a reassembly operator. The encoder aggregates local content into a lower-resolution feature, the kernel predictor generates position-specific upsampling kernels, and the reassembly operator uses these kernels to reconstruct a higher-resolution feature map. In the neck, CARAFE replaces fixed interpolation with a content-aware upsampling operator. Instead of using the same weights everywhere, CARAFE looks at the local neighborhood around each position, predicts a small reassembly kernel conditioned on the local content, and then uses this kernel to reconstruct the upsampled feature map. This helps preserve fine edges and tiny defect patterns when going from low-resolution to high-resolution features. Formally, given a low-resolution feature map $X\in {\mathbb{R}}^{C\times H\times W}$ and upsampling factor r , CARAFE first encodes local content:

(4) $$E=H_{\mathrm{enc}}(X),$$

and predicts spatially varying reassembly kernels by

(5) $$K=H_{\ker}(E).$$

where K stores a reassembly kernel for each spatial location. Let unfold(X) denote the unfolded local patches of X. For each low-resolution location p and subpixel offset δ (corresponding to a position in the r×r upsampled neighborhood), the high-resolution output Y is reconstructed as:

(6) $$Y(p,\delta )=\sum_{t\in N(p)}K(p,\delta ,t)\cdot X(t).$$

where N(p) is the local neighborhood around p. In the top-down path (P5→P4, P4→P3), CARAFE replaces fixed interpolation, preserving fine edges and small patterns with minimal overhead.

In the top-down path (P5→P4, P4→P3), CARAFE replaces fixed interpolations, preserving fine edges and small patterns with minimal overhead. As shown in Figure 3, CARAFE upsamples a feature map by first encoding local content and then predicting position-adaptive reassembly kernels.

#### 2.1.4. BiFPN-Style Fusion with WA_SC (WeightedAdd + Separable Convolution)

**Figure 4:**
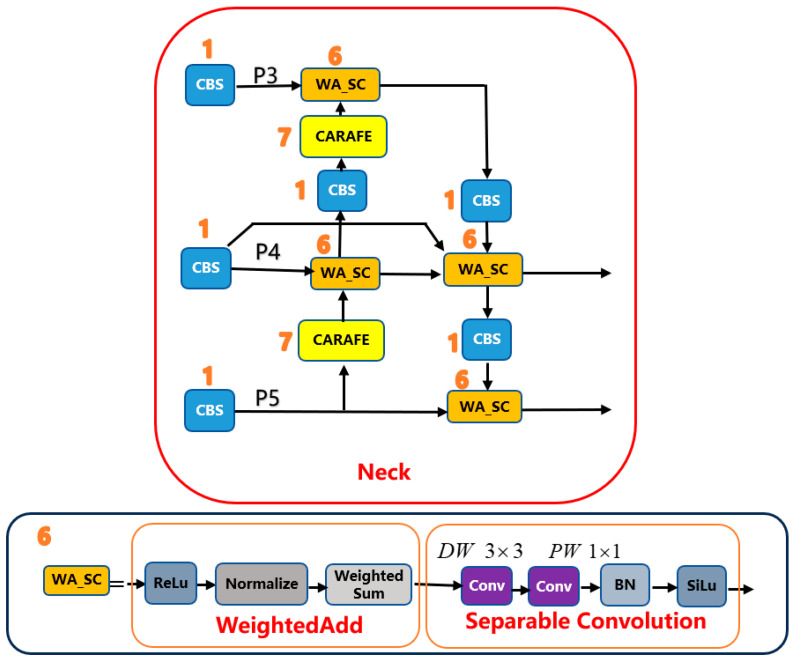
BiFPN-style multi-scale fusion with the WA_SC node. Each fusion node takes several input features at different resolutions, normalizes learnable fusion weights across inputs, forms a weighted sum, and refines the result with a depthwise separable convolution. The number ‘6’ indicates the WA_SC module.

Each BiFPN fusion node, depicted in Figure 4, can be described by three steps: (i) assign a non-negative learnable weight to each input feature; (ii) normalize these weights so that they sum to 1; and (iii) form a weighted sum of the inputs followed by a depthwise separable convolution. Let $\left\{x_{i}\right\}$ be the inputs and $\left\{w_{i}\right\}$ the raw fusion weights. For inputs $\left\{x_{i}\right\}$ entering a fusion node at a fixed scale, we learn a scalar weight $\left\{w_{i}\right\}$ for each input and normalize them as:

(7) $${\hat{w}}_{i}=\frac{w_{i}}{\varepsilon +\sum_{j}w_{j}}$$

where ε is a small constant for numerical stability. The fused tensor is then computed as:

(8) $$Y=\sum_{i}{\hat{w}}_{i}\cdot X_{i}$$

and refined by a depthwise-separable convolution block consisting of a depthwise 3 × 3 convolution (stride 1, padding 1, groups = C) followed by a pointwise 1 × 1 convolution (stride 1), both with Batch Normalization and a SiLU activation.

We refer to this pair—the normalized WeightedAdd followed by the depthwise-separable refinement—as the WA_SC block. An identical WA_SC block is used at every BiFPN fusion node in both the top-down (P5→P4→P3) and bottom-up (P3→P4→P5) paths. Before fusion, P5 and P4 features are projected to 640 channels and P3 to 320 channels by 1 × 1 lateral convolutions so that all inputs to a WA_SC node have matching channel dimensions (see Appendix A for the exact configuration).

The detailed internal structure of the BiFPN neck and its WA_SC fusion nodes is illustrated in Figure 4, where each fusion node explicitly shows the successive WeightedAdd and depthwise-separable convolution operations. Figure 4 gives a schematic view of the bidirectional BiFPN neck and its WA_SC fusion nodes.

#### 2.1.5. DCN-Lite on the High-Resolution Path (P3)

**Figure 5:**
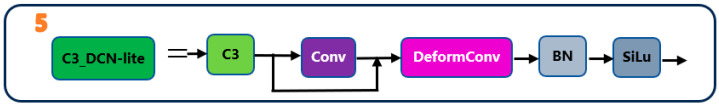
DCN-lite module on the P3 (stride-8) feature map. A small convolution predicts offsets for a 3 × 3 sampling grid, and the shifted grid is used to perform deformable convolution on P3, enhancing geometric flexibility for small defects. The number ‘5’ indicates the C3_DCN-lite module.

The DCN-lite block on P3, illustrated in Figure 5, predicts a small offset for each position of a 3 × 3 sampling grid and then uses the shifted grid to perform convolution. This gives the highest-resolution feature map mild geometric flexibility to better fit irregular defect shapes. Formally, a single DCN-lite is applied to the P3 branch (stride 8) to introduce localized geometric flexibility with minimal latency overhead. For an input feature map X and learned offsets $\left\{\Delta p_{i}\right\}$ on the sampling grid $R\;=\;\{p_{i}\}$ (e.g., a 3 × 3 grid), the deformable convolution output at location $p_{0}$ is

(9) $$Y(p_{0})=\sum_{p_{i}\in R}w(p_{i})\cdot X(p_{0}+p_{i}+\Delta p_{i}),$$

where $w(p_{i})$ are the convolution weights and bilinear interpolation is used for non-integer sampling coordinates. Concentrating the deformable operation at the finest resolution (P3) enhances sensitivity to small and irregular defects while keeping the model lightweight. The placement and structure of the DCN-lite module on the P3 feature map are depicted in Figure 5.

### 2.2. One-Stage Semi-Supervised Training

The training scheme exploits unlabeled images via a single-cycle pseudo-label self-training pipeline with three stages. We first train the detector on the small labeled set, then run it on the unlabeled images to obtain high-confidence predictions as pseudo-labels, and finally retrain the model on the union of labeled and pseudo-labeled data. This design intentionally keeps the semi-supervised part simple and implementation-friendly. No EMA teacher, test-time augmentation (TTA), or labeled/unlabeled sampling-ratio heuristics are used. Compared with more advanced teacher–student SSOD frameworks such as Unbiased Teacher, Soft Teacher, or DenseTeacher, which rely on EMA teachers, strong/weak augmentation pairs, and extra consistency or dense pseudo-labeling losses, our design is intentionally minimalist and aims to provide a lightweight, implementation-friendly baseline that can be readily plugged into existing YOLOv5-based AOI pipelines on PCB images.

#### 2.2.1. Notation

Let the labeled and unlabeled sets be

(10) $$L=\left\{\left(x_{l},y_{l}\right)\right\},\;U=\left\{x_{u}\right\}.$$

For an image x, the detector outputs a set of predicted bounding boxes $\left\{b_{j}\right\}$, class posteriors $\left\{p_{j}\right\}$, and objectness scores $\left\{o_{j}\right\}$, The detection confidence for prediction j is defined as

(11) $${\mathrm{conf}}_{j}=o_{j}\cdot \max_{c}p_{j,c}.$$

#### 2.2.2. Stage A—Supervised Pre-Training

Stage A is standard supervised training on the labeled set L. We use the Full model as the detector and optimize the usual YOLOv5 detection loss, which combines bounding-box regression, objectness and classification terms over the three detection layers. The loss for a mini-batch B⊂L can be written as:

(12) $$L_{\det}(B;\theta )=\lambda_{\mathrm{box}}L_{\mathrm{box}}+\lambda_{\mathrm{obj}}L_{\mathrm{obj}}+\lambda_{\mathrm{cls}}L_{\mathrm{cls}}$$

where θ denotes the model parameters. We use the YOLOv5 default gains $\left(\lambda_{\mathrm{box}},\lambda_{\mathrm{obj}},\lambda_{\mathrm{cls}})=(0.05,1.0,0.5\right)$. $L_{\mathrm{box}}$ is the bounding-box regression loss (e.g., CIOU) computed on positive anchors, $L_{\mathrm{obj}}$ is the BCE objectness loss with IoU-based soft targets for positives and 0 for negatives, and $L_{\mathrm{cls}}$ is the BCE classification loss with optional label smoothing. Layer-balance coefficients for the three detection layers also follow the YOLOv5 defaults.

#### 2.2.3. Stage B—Pseudo-Label Generation

In Stage B, we run the Stage-A model on every unlabeled image and convert confident detections into pseudo-labels. Using the trained Full model, inference is run over all $x_{u}\in U$. After non-maximum suppression, only predictions whose confidence exceeds a fixed threshold τ are kept as pseudo-annotations:

(13) $$\hat{U}=\left\{(x_{u},{\hat{y}}_{u}):x_{u}\in U{\;\mathrm{and}\;\mathrm{conf}}_{j}(x_{u})\geq \tau \right\}$$

The threshold τ is fixed throughout all experiments.

#### 2.2.4. Stage C—Retraining on the Mixed Pool (Uniform Sampling)

The mixed training set is

(14) $$T=L\cup \hat{U}$$

In Stage C, mini-batches are sampled uniformly at random from T without any labeled/unlabeled ratio control. The objective is to minimize the same detection loss over T, treating pseudo-labels and ground-truth labels identically:

(15) $$\min_{\theta }E_{(x,y)\sim T}\left[L_{\det}(x,y;\theta )\right]$$

Here (x, y) may correspond to either a labeled example from L or a pseudo-labeled sample from $\hat{U}$.

#### 2.2.5. Flowchart and Implementation Remarks

Figure 6 summarizes the single-cycle semi-supervised training pipeline described in Section 2.2.1, Section 2.2.2, Section 2.2.3 and Section 2.2.4 Stage A trains the Full model on the labeled set to obtain an initial detector; Stage B runs inference on the unlabeled pool and retains high-confidence predictions as pseudo-labels; Stage C retrains the detector on the mixed labeled and pseudo-labeled pool using the same detection loss.

Figure 6 visualizes the single-cycle procedure. Stage A trains on labeled data to obtain the initial detector; Stage B generates pseudo-labels on U by confidence thresholding; Stage C retrains on T with uniform sampling and the same detection loss for both label sources.

Implementation details.

- Sampling. Mini-batches are drawn uniformly at random from the mixed set $L\cup \hat{U}$; no labeled/unlabeled ratio is enforced.
- Confidence thresholding & NMS. A single, class-agnostic confidence threshold τ is used for pseudo-label generation (Stage B). The NMS configuration (e.g., IoU threshold) matches that which is used in validation for consistency.
- Loss & assignment. The detection loss and anchor/assignment strategy follow the standard YOLOv5 implementation. Accepted pseudo-labels are treated as hard targets in Stage C, identical to ground-truth annotations.

### 2.3. Summary of Design Choices

Rather than proposing entirely new backbone or neck primitives, our design adopts a minimalist strategy: it selects a small set of proven modules and places them where they are most beneficial for tiny PCB defects and label-scarce training. The key design choices are:

Channel re-weighting: ECA inside C3 improves feature selectivity with negligible overhead (see Section 2.1.2).

Content-aware upsampling: CARAFE preserves fine structures while injecting context into high-res maps (Section 2.1.3).

Cross-scale fusion: BiFPN-style WeightedAdd provides normalized, non-negative fusion that balances semantics and detail (Section 2.1.4).

Geometric flexibility: A single P3 DCN-lite targets the finest scale, improving small-defect recall at low latency (Section 2.1.5).

Semi-supervision: A one-cycle pseudo-labeling scheme with uniform sampling serves as a lightweight SSOD baseline that avoids EMA teachers, strong/weak augmentation pairs, or labeled/unlabeled ratio heuristics, yet still yields measurable gains under label-scarce PCB conditions (Section 2.2).

For exact reproducibility, the full YOLOv5-ECA-DCN-BiFPN-CARAFE configuration file used in all experiments is provided as Appendix A in the Appendix, specifying every backbone and neck layer, including kernel sizes, strides and channel dimensions.

### 2.4. Data and Training Setup

Experiments are conducted on the PKU-PCB [50] dataset comprising six defect categories (open circuit, short circuit, mouse bite, spur, spurious copper, and pin-hole). To reflect realistic production constraints—few labeled images and many unlabeled images—the training data are split into a small labeled subset and a larger unlabeled pool, with independent validation and test sets held out.

The default semi-supervised configuration, which matches the dataset-split schematic, is summarized in Table 1.

All subsets are disjointed (no image overlap). Unlabeled images contribute to training only via pseudo-labels; the test set remains completely unseen until the final evaluation. The validation (600) and test (2134) splits remain fixed and non-overlapping across all experiments. This design enables controlled comparisons across label-scarce regimes while preserving a consistent, independent benchmark for model selection and final reporting. In the 100 labeled training images used for supervised and semi-supervised learning, there are 215 annotated defects in total: 36 open, 36 short, 34 mouse bite, 38 spur, 40 spurious copper, and 31 pin-hole instances. The held-out test set of 2134 images contains 4349 annotated defects, with 660, 706, 803, 765, 676 and 739 instances for the six classes, respectively. Thus, each class accounts for roughly 14–19% of all annotations. The dataset is therefore not extremely long-tailed, but the labeled subset still provides somewhat fewer examples for certain categories (e.g., mouse bite and pin-hole) than for others, which can make these defects harder to learn reliably from labeled data alone.

In this work, all experiments are conducted on the PKU-PCB dataset, which we adopt as a representative public benchmark for multi-class PCB defect detection. We acknowledge that restricting evaluation to a single dataset limits the assessment of cross-dataset generalizability, and we therefore interpret our findings as evidence of effectiveness on PKU-PCB rather than as universal conclusions for all PCB settings.

Training Details. All experiments were conducted on a workstation equipped with a NVIDIA RTX 4090 (24 GB) GPU, an Intel^®^ Xeon^®^ Gold 6258R CPU, and 128 GB RAM. The software environment comprised Python 3.10 and PyTorch 2.1, running the official YOLOv5 codebase. Models were trained with a batch size of 16 using SGD (momentum 0.937, weight decay 5 × 10^−4^). The initial learning rate was 0.01 and followed a cosine decay schedule over the course of training. Each phase was run for up to 200 epochs, with early stopping triggered by a plateau in validation mAP to mitigate overfitting. Standard YOLOv5 data augmentations were enabled—random image scaling, horizontal flipping, color jitter, and Mosaic composition—to increase appearance diversity and improve generalization under limited labels. During inference, the confidence threshold was 0.25 (YOLOv5 default). For pseudo-label generation in the semi-supervised stage, a stricter threshold τ = 0.60 was applied to retain only high-confidence detections for retraining.

## 3. Results

### 3.1. Evaluation Metrics

Detection quality is reported with precision (P), recall (R), average precision (AP), and the mean AP at IoU = 0.5 (mAP@0.5). Model size (Params) and computation (GFLOPs) are provided for complexity.

IoU. A prediction $\hat{b}$ matches a ground-truth box b if

(16) $$\mathrm{IoU}(\hat{b},b)=\frac{\left|\hat{b}\cap b\right|}{\left|\hat{b}\cup b\right|}\geq 0.5,$$

Precision and Recall. With true positives (TP), false positives (FP), and false negatives FN,

(17) $$P=\frac{TP}{TP+FP},\;R=\frac{TP}{TP+FN},$$

High P indicates few false alarms; high R indicates few missed defects.

AP. For each class c, detections are sorted by confidence to form a precision–recall curve $P_{c}(R)$. The class AP is the area under this curve:

(18) $${\mathrm{AP}}_{c}=\int_{0}^{1}P_{c}(R)dR,$$

(implemented as the discrete, interpolated sum over recall breakpoints).

mAP@0.5. The primary metric averages AP across all C defect classes at the fixed IoU threshold 0.5:

(19) $$\mathrm{mAP}@0.5=\frac{1}{C}\sum_{c=1}^{C}{\mathrm{AP}}_{c}{|}_{\mathrm{IoU}=0.5}.$$

Class-wise reporting. For each class, P, R, and $AP_{c}$ are computed in a one-vs-all manner to reveal class difficulty; mAP summarizes overall performance.

Complexity. Params is the total number of learnable weights. GFLOPs denotes the number of giga floating-point operations per image at test time, estimating computational cost.

### 3.2. Results and Analysis

#### 3.2.1. Overall Performance and Module Ablation (Supervised)

Unless otherwise specified, all results reported in this section are obtained on the PKU-PCB test set under the label-scarce setting described in Section 2.4. The baseline YOLOv5 model is first evaluated against progressively enhanced variants under fully supervised training (using the available labeled training set) to quantify the impact of each architectural module. Table 1 summarizes the results. The baseline YOLOv5 achieves 87.0% mAP@0.5 on the PCB test set, with precision 91.9% and recall of 83.5%. This high baseline underscores YOLOv5’s strong starting point for PCB defect detection. Adding the Efficient Channel Attention block (+ECA) to YOLOv5 yields a slight mAP improvement to 88.0%, indicating better channel-wise feature focus on subtle defects. Incorporating a lightweight deformable convolution layer (+ECA+DCN) further raises mAP to 88.9% and improves recall (83.5%→84.8%), confirming that spatially adaptive convolution helps detect a few more irregularly shaped defects. Replacing the standard upsampling with the CARAFE operator (+ECA+DCN+CARAFE) gives another modest boost (mAP 89.3%, recall of 86.3%), suggesting better preservation of fine-grained features for small defect regions. The largest gain comes from introducing the BiFPN neck (+ECA+DCN+CARAFE+BiFPN), which produces our Full model (the proposed ECA–DCN-lite–BiFPN–CARAFE-enhanced YOLOv5). The Full model reaches 91.0% mAP@0.5 with precision 93.5%. The BiFPN’s stronger multi-scale feature fusion substantially improves detection of tiny, low-contrast defects, reflected in the higher overall accuracy. Notably, the Full model attains the highest mAP and also the highest precision among all variants, indicating that it not only finds more defects but also triggers fewer false alarms. 

In addition to accuracy, our proposed modifications improve efficiency. Despite integrating several new modules, the Full model is actually lighter than the baseline YOLOv5 (parameters reduced from 86.2 M to 63.9 M) and requires fewer operations per inference (175 GFLOPs vs. 203.9 GFLOPs). This reduction is due to replacing some heavy layers with streamlined ones (e.g., using SPPF-lite and separable convolutions) and the BiFPN’s optimized topology. In summary, each architectural module contributed incremental gains, and together they delivered an overall +4.0 mAP point improvement (87.0→91.0) over the baseline (Table 2). Table 2 summarizes precision, recall, mAP@0.5, parameters, and GFLOPs for all supervised variants on the PKU-PCB test set. The proposed model (ECA–DCN-lite–BiFPN–CARAFE-enhanced YOLOv5) achieves state-of-the-art performance on this task under full supervision.

Beyond the internal ablations, we also compare the proposed detector with several recent state-of-the-art YOLO-based methods used for defect or small-object detection. Table 2 also includes YOLOv8 [51] and three recent improved YOLO-based detectors from the literature (YOLOv9–YOLOv11) [52,53,54]. The official YOLOv8 (with a model size of ~68 M) reached 87.4% mAP on our PCB test, slightly above the YOLOv5 baseline. YOLOv9 and YOLOv11 (from published methods) achieved around 88.8–88.9% mAP, indicating incremental improvements over YOLOv5 but still falling short of our Full model. Notably, our Full model outperforms all of these, with 91.0% mAP@0.5, establishing a new state-of-the-art for PCB defect detection under the same training conditions. It is worth mentioning that the Full model’s accuracy advantage comes without excessive model complexity—for instance, YOLOv9/YOLOv11 have slightly fewer parameters (≈58 M) but still lower accuracy (∼88.8% mAP), while YOLOv8 is both larger and less accurate than our model. This highlights that our combination of ECA, DCN-lite, CARAFE, and BiFPN is particularly effective for the unique challenges of PCB inspection (very small defects and complex backgrounds), yielding superior accuracy without undue model bloat. In other words, the targeted design of our modules provides a bigger payoff on this specialized task than generic YOLO improvements.

Transformer-based detectors, such as DETR and RT-DETR-style models, as well as PCB-oriented Transformer–YOLO variants, have also shown strong performance in recent literature. However, these methods usually require substantial pre-training on large general-purpose datasets (e.g., COCO), longer training schedules, and heavier computation or memory footprints. In this study we therefore focus our empirical comparison on the YOLO family, using a unified training pipeline on PKU-PCB, and position our ECA–DCN-lite–BiFPN–CARAFE design as a label-efficient, deployment-oriented enhancement to YOLOv5. A thorough experimental comparison with representative Transformer-based detectors under the same label-scarce PCB setting is left as important future work.

#### 3.2.2. Training Convergence Across Architectures

**Figure 7:**
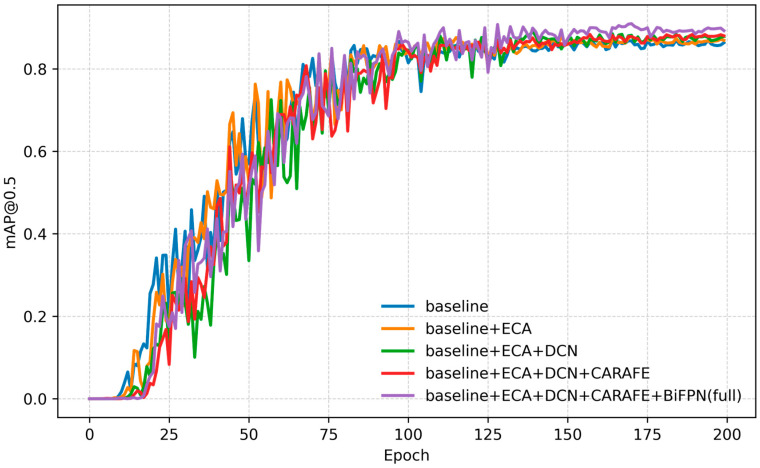
Training curves of mAP@0.5 versus epoch for the baseline and four enhanced architectures.

Training curves of mAP@0.5 versus epoch number for the five model variants are plotted in Figure 7. All models exhibit a rapid initial rise in accuracy over the first ~20–30 epochs, followed by a more gradual improvement up to ~120 epochs, and then a plateau as training converges. Importantly, at any given epoch the curve for a model with an extra module lies at or above the curve for the less advanced model, indicating strictly monotonic improvements from each architectural addition. The Baseline (YOLOv5) saturates at the lowest mAP (~0.87), whereas adding ECA shifts the entire learning trajectory upward (even the early epochs start higher), reflecting better channel-attention focus on relevant features. Adding the DCN-lite accelerates mid-phase learning (steeper gain around 40–80 epochs) consistent with improved geometric flexibility. Replacing nearest-neighbor upsampling with CARAFE yields a modest but steady uplift and also smooths out minor fluctuations near convergence, suggesting more stable feature interpolation. Finally, the Full model (with BiFPN) not only reaches the highest final mAP (~0.91) but also converges faster than the others, with its curve separating from the pack early on. By epoch ~100, the Full model virtually attains its peak, whereas the Baseline still creeps up slowly. This indicates that the enhanced architecture learns more efficiently from limited data. Throughout training, the performance ranking remains consistent—Baseline < +ECA < +ECA+DCN < +ECA+DCN+CARAFE < Full—mirroring the final test-set results in Table 1. In practical terms, these dynamics mean that our Full model can achieve a given accuracy in fewer epochs, which is advantageous for faster experimentation and deployment.

#### 3.2.3. Semi-Supervised Learning Results

Semi-Supervised vs. Supervised Training: Leveraging unlabeled data via semi-supervised learning (SSL) led to substantial performance gains. Table 3 compares the final detector’s results after semi-supervised training with the corresponding supervised-only results. Using the baseline YOLOv5 architecture, adding the SSL procedure (with 100 labeled images and 1000 unlabeled images in training) improved mAP from 87.0% to 91.15%, and increased recall from 83.5% to 87.4%. This indicates that pseudo-labeling the additional 1000 PCB images effectively enhanced the model’s ability to catch more defects (higher recall) without sacrificing precision (which also rose to 93.6%). For the Full model proposed architecture, semi-supervised training provided an even larger boost: the mAP climbed from 91.0% to 94.3%, with precision 94.4% and recall of 91.2%. In other words, our model trained with unlabeled data achieves 94.3% detection mAP@0.5—an absolute improvement of ~3.3 points over the supervised version and ~7.3 points over the supervised baseline. These results validate that the one-stage SSL strategy markedly improves defect detection performance, especially by reducing false negatives (since recall gains are most pronounced). The extra unlabeled examples expose the detector to a wider diversity of PCB appearances and defect instances, mitigating overfitting to the small labeled set. These semi-supervised gains are summarized in Table 3 for both the baseline YOLOv5 and the Full model.

An additional advantage of our approach is its label efficiency. With the semi-supervised pipeline, very high accuracy can be achieved even with only a small fraction of the data labeled. For instance, our Full + SSL model (proposed model with semi-supervised training) can exceed 90% mAP with only the order of dozens of labeled images, by learning from hundreds of unlabeled samples via pseudo-labeling (a scenario that would yield far lower accuracy if trained supervised-only). This data-efficient learning is extremely valuable in real production settings where labeling is expensive. In summary, leveraging unlabeled data via SSL markedly improves detection—especially by increasing recall—and our results show this holds across different label budgets. Relation to advanced SSOD methods. Recent semi-supervised object detection frameworks such as Unbiased Teacher, Soft Teacher, DenseTeacher, and DTG-SSOD have demonstrated impressive gains on generic benchmarks (e.g., MS COCO) by combining EMA teacher–student architectures with strong/weak augmentation schemes, consistency regularization, or dense pseudo-labels. While powerful, these methods introduce additional networks, losses, and hyperparameters, and typically require longer training schedules and larger-scale datasets, which complicates deployment in resource- and engineering-constrained AOI environments. In contrast, our one-stage pseudo-labeling pipeline is a simple add-on to the standard YOLOv5 training script and already yields substantial improvements: +4.15 mAP@0.5 (87.0→91.15) for the baseline YOLOv5 and +3.3 mAP@0.5 (91.0→94.3) for the Full model, with a total gap of 7.3 mAP@0.5 over the supervised baseline. We therefore position our SSL component as a practical, deployment-oriented baseline for PCB defect detection. A promising direction for future work is to adapt teacher–student SSOD frameworks to PCB AOI and to investigate whether their added complexity brings further benefits under industrial constraints.

#### 3.2.4. Class-Wise Detection Performance

To understand performance per defect type, Table 4 breaks down the precision, recall, and AP@0.5 for each of the six PCB defect classes, comparing the baseline, the Full model, and the Full model with SSL. The Full model provides consistent improvements over the baseline for all defect categories, and incorporating unlabeled data (Full + SSL) further boosts performance, most prominently in recall. For example, for the “short circuit” defect (which the baseline found relatively challenging), the baseline model obtains AP = 80.7% with recall of 77.6%. The Full model improves this to AP = 87.6% and recall of 82.7%, and with Full + SSL model it reaches AP = 89.8% and recall of 88.8%, substantially reducing missed short-circuit defects. Similar trends are observed in other classes: for “spur” defects, AP rises from 83.0%→87.4%→92.4% moving from baseline to Full to Full + SSL, driven by recall improving from 76.4%→77.6%→86.5%. Even for classes where baseline precision was already very high (e.g., “pin-hole”, P ≈ 98%), the Full model and SSL manage to increase recall (baseline 92.4%→Full 95.8%→Full + SSL 97.9%) and achieve the highest AP (98.5%). These results indicate that our proposed modules and semi-supervised training generalize across defect types—every category sees an increase in detection quality, and the greatest gains come in catching difficult instances (recall). The Full + SSL model does especially well on defects that are tiny or low-contrast (e.g., spurious copper, pin-holes), where it dramatically reduces false negatives compared to the baseline. Overall, the class-wise analysis confirms that the improved detector is robust across all defect categories, offering both higher sensitivity and precision than the original YOLOv5.

As summarized in Section 2.4, all six defect classes have comparable frequency on PKU-PCB (each between about 14% and 19% of all annotations), but the 100 labeled training images still contain fewer manual examples for some categories such as mouse bite (34 instances) and pin-hole (31 instances) compared with, for example, spurious copper (40 instances). Despite this mild imbalance and the varying shape and scale of the defects, the proposed Full and Full + SSL models improve AP@0.5 and recall for all six classes in Table 4. In particular, the classes with fewer labeled samples in the training set (mouse bite and pin-hole) also benefit from the architectural enhancements and the pseudo-label-based SSL, suggesting that the method helps reduce missed detections of these relatively under-represented defects.

#### 3.2.5. Qualitative Results

Overall, the proposed model substantially improves detection performance on PKU-PCB under label-scarce regimes. With supervised training only, mAP@0.5 increases from 0.870 (baseline YOLOv5) to 0.910 while reducing parameters and FLOPs. When combined with the single-cycle semi-supervised scheme, the proposed model reaches 0.943 mAP@0.5 with 94.4% precision and 91.2% recall, outperforming both the baseline and recent YOLO variants.

**Figure 8:**
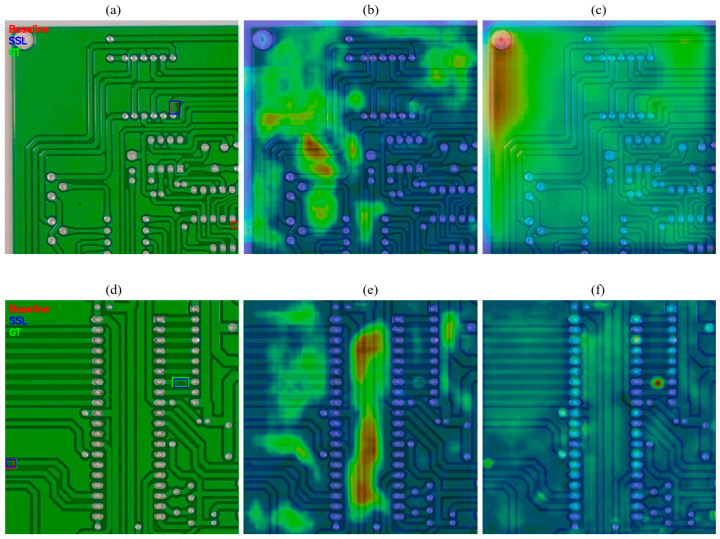
Qualitative detection and attention-like feature visualizations on PKU-PCB. (**a**) Detection results of a normal PCB board by the Baseline and Full + SSL models compared to the Ground Truth (GT). (**b**) P3-level heatmap of the Baseline model corresponding to (**a**). (**c**) P3-level heatmap of the Full + SSL model corresponding to (**a**). (**d**) Detection results of another normal PCB board by the Baseline and Full + SSL models compared to the Ground Truth (GT). (**e**) P3-level heatmap of the Baseline model corresponding to (**d**). (**f**) P3-level heatmap of the Full + SSL model corresponding to (**d**).

As shown in Figure 8, the top row (a–c) shows a board region that contains no defect. In (a), the Baseline YOLOv5 (red box) incorrectly predicts a spur-like defect, whereas the proposed Full + SSL model (blue) produces no false alarm, consistent with the ground truth (green). Panels (b) and (c) show the corresponding P3 (stride-8) feature heatmaps for the Baseline and Full + SSL models, respectively. The heatmaps visualize the L2-norm of the P3 feature responses overlaid on the input image. The Baseline exhibits broad, noisy activations along the traces, while the Full + SSL model yields a more uniform response that does not highlight non-defect regions.

The bottom row (d–f) shows a board region containing a small mouse bite defect. In (d), the Baseline fails to produce a correct detection at the annotated location, effectively missing the defect, whereas the Full + SSL model predicts a tight box aligned with the ground truth. The corresponding P3 heatmaps in (e) and (f) reveal that the Baseline still has diffuse responses that do not emphasize the defect, while the Full + SSL model presents a strong, localized activation exactly at the defect site. These examples visually confirm the improvements in precision and recall achieved by the proposed architecture and semi-supervised training.

### 3.3. Reproducibility and Variance Analysis

To evaluate the robustness of the reported gains, we trained the four key configurations—Baseline, Full, Baseline + SSL and Full + SSL—with six different random seeds and recorded the resulting mAP@0.5 on the PKU-PCB test set. The scores and their statistics are summarized in Table 5.

The standard deviations are small for all models (0.004–0.012), indicating that the training procedure is numerically stable. The supervised Baseline and Full model achieve mean mAP@0.5 values of 0.874 and 0.911, respectively, while the semi-supervised Baseline + SSL and Full + SSL models obtain 0.906 and 0.936. Thus, the Full model consistently outperforms the Baseline by about 3.7 percentage points in mAP@0.5, and the Full + SSL model improves over Baseline + SSL by about 3.0 percentage points. These gaps are substantially larger than the observed variance, confirming that the accuracy improvements brought by the architectural modifications and by the pseudo-label-based SSL scheme are robust to random initialization and data shuffling. The mean values are also close to the single-run results listed in Table 2, Table 3 and Table 4, showing that those reported numbers are representative of typical runs.

## 4. Conclusions

This paper presented a label-efficient PCB defect detector that augments YOLOv5 with ECA, DCN-lite, a BiFPN neck with WeightedAdd and separable convolutions, and CARAFE upsampling, together with a single-cycle semi-supervised training scheme. On the PKU-PCB benchmark, the proposed ECA–DCN-lite–BiFPN–CARAFE-enhanced YOLOv5 improves supervised mAP@0.5 from 0.870 (baseline YOLOv5) to 0.910 while reducing parameters and GFLOPs. When combined with semi-supervised training on 100 labeled and 1000 unlabeled images, the Full + SSL model further reaches 0.943 mAP@0.5 with 94.4% precision and 91.2% recall, outperforming several recent YOLO-based detectors and delivering consistent gains across all defect classes.

The main advantages of the proposed design are: (i) higher accuracy for tiny, low-contrast PCB defects due to targeted architectural modules; (ii) strong label-efficiency enabled by the one-stage pseudo-labeling pipeline; and (iii) moderate model size and computation, which facilitate deployment in AOI systems. These strengths make the approach attractive for practical PCB inspection scenarios where annotation budgets are limited. A small variance analysis over multiple random seeds further confirms that the observed mAP gains are stable and not due to a single favorable run.

Nonetheless, this work has several limitations. First, all experiments are conducted on a single public PCB dataset (PKU-PCB), so the generalizability of the gains to other PCB designs, imaging setups, and industrial lines remains to be verified. Second, the study focuses on a single detector family (YOLO-based one-stage detectors, instantiated here as YOLOv5) and explores only a basic one-cycle pseudo-labeling scheme, without numerical comparison to more advanced teacher–student SSOD frameworks such as Soft Teacher or Unbiased Teacher, or to Transformer-based detectors such as DETR or RT-DETR variants. Although the proposed detector substantially improves detection performance for all six defect types under a moderately imbalanced label distribution, residual gaps between classes remain, and future work will explore explicit imbalance-aware strategies such as class rebalancing or cost-sensitive losses to further enhance recognition of relatively less frequent or safety-critical defects.

Future research will extend the evaluation to additional PCB and industrial defect datasets, explore other backbone/neck variants and more advanced semi-supervised strategies, and assess the method in end-to-end AOI systems on real production lines. It will also benchmark the proposed architecture against representative Transformer-based models under the same label-efficient AOI constraints and investigate combining Transformer-style global context with the proposed YOLOv5 enhancements.

Overall, the contribution of this work is primarily system- and application-oriented: it shows that a carefully designed combination of existing attention, deformable and feature-fusion modules, together with a simple single-cycle semi-supervised scheme, can deliver substantial gains for tiny, low-contrast PCB defects under realistic label constraints. Future research will explore extending these design principles to other detector families and more advanced semi-supervised strategies.

By enabling accurate, efficient, and scalable AI-driven PCB inspection for modern production lines, this work directly advances SDG 9: Industry, Innovation and Infrastructure, supporting smart manufacturing and resilient industrial automation.

## Figures and Tables

**Table 1:** PKU-Dataset Splits and Usage Across Training Phases.

Subset	Images	Purpose
Labeled train	100	Used for Phase I supervised training (initial model)
Unlabeled pool	1000	Used for Phase II pseudo-label generation in semi-supervised training
Validation	600	Model selection, hyperparameter tuning, and early stopping
Test	2134	Held-out evaluation of final performance (not used in training)

**Table 2:** Detection performance on the PKU-PCB test set (supervised, 100 labeled images).

Model	Stage	P	R	mAP@0.5	Params	GFLOPs
YOLOv5 (baseline)	supervised	0.919	0.835	0.870	86.21 M	203.86
+ECA	supervised	0.906	0.844	0.880	86.21 M	203.88
+ECA+DCN	supervised	0.932	0.848	0.889	87.13 M	215.69
+ECA+DCN+ CARAFE	supervised	0.917	0.863	0.893	88.50 M	217.50
+ECA+DCN+ CARAFE +BiFPN (Full)	supervised	0.935	0.847	0.910	63.91 M	175.51
YOLOv8	supervised	0.914	0.812	0.874	68.16 M	258.15
YOLOv9	supervised	0.928	0.816	0.888	58.15 M	192.70
YOLOv10	supervised	0.890	0.778	0.860	31.67 M	171.05
YOLOv11	supervised	0.910	0.816	0.889	56.88 M	195.48

**Table 3:** Effect of semi-supervised learning with 100 labeled and 1000 unlabeled images.

Model	P	R	mAP@0.5
Baseline + SSL	0.936	0.874	0.915
Full + SSL	0.944	0.912	0.943

**Table 4:** Class-wise detection results (AP@0.5, precision, recall) for the Baseline, Full, and Full + SSL models on the PKU-PCB test set.

Defect Class	Baseline P	Baseline R	Baseline mAP@0.5	Full P	Full R	Full mAP@0.5	Full + SSL P	Full + SSL R	Full + SSL mAP@0.5
open	0.843	0.883	0.907	0.962	0.889	0.943	0.937	0.945	0.962
short	0.919	0.776	0.807	0.918	0.827	0.876	0.915	0.888	0.898
mouse bite	0.954	0.834	0.881	0.967	0.800	0.895	0.975	0.881	0.941
spur	0.934	0.764	0.830	0.950	0.776	0.874	0.939	0.865	0.924
copper	0.885	0.828	0.840	0.882	0.833	0.904	0.948	0.916	0.948
Pin-hole	0.980	0.924	0.957	0.930	0.958	0.969	0.949	0.979	0.985

**Table 5:** Reproducibility and variance analysis of mAP@0.5 over N independent runs.

Model	Run 1 mAP@0.5	Run 2 mAP@0.5	Run 3 mAP@0.5	Run 4 mAP@0.5	Run 5 mAP@0.5	Run 6 mAP@0.5	Mean mAP@0.5	Std. Dev.
Baseline	0.870	0.883	0.872	0.869	0.868	0.881	0.874	0.006
Full	0.910	0.904	0.916	0.911	0.909	0.917	0.911	0.004
Baseline + SSL	0.915	0.908	0.917	0.896	0.888	0.914	0.906	0.012
Full + SSL	0.943	0.933	0.930	0.938	0.938	0.934	0.936	0.005

## Data Availability

The data are contained within the article.

## Appendix A

The following YAML file specifies the full training configuration of the proposed ECA–DCN-lite–BiFPN–CARAFE-enhanced YOLOv5 detector for the PKU-PCB dataset (nc = 6):

nc: 6

depth_multiple: 1.33

width_multiple: 1.25

anchors:

- [10, 13, 16, 30, 33, 23]

- [30, 61, 62, 45, 59, 119]

- [116, 90, 156, 198, 373, 326]

backbone:

- [−1, 1, Conv, [64, 6, 2, 2]]

- [−1, 1, Conv, [128, 3, 2]]

- [−1, 3, C3ECA, [128]]

- [−1, 1, Conv, [256, 3, 2]]

- [−1, 6, C3ECA, [256]]

- [−1, 1, Conv, [512, 3, 2]]

- [−1, 9, C3ECA, [512]]

- [−1, 1, Conv, [1024, 3, 2]]

- [−1, 3, C3, [1024]]

- [−1, 1, SPPFLite, [1024, 5]]

BiFPN head:

- [−1, 1, Conv, [640, 1, 1]]

- [6, 1, Conv, [640, 1, 1]]

- [4, 1, Conv, [320, 1, 1]]

- [10, 1, CARAFE, [2]]

- [[11, 13], 1, WeightedAdd, [2]]

- [14, 1, SeparableConv, [3, 1]]

- [15, 1, Conv, [320, 1, 1]]

- [16, 1, CARAFE, [2]]

- [[12, 17], 1, WeightedAdd, [2]]

- [18, 1, SeparableConv, [3, 1]]

- [19, 1, Conv, [640, 3, 2]]

- [[11, 15, 20], 1, WeightedAdd, [3]]

- [21, 1, SeparableConv, [3, 1]]

- [22, 1, Conv, [640, 3, 2]]

- [[10, 23], 1, WeightedAdd, [2]]

- [24, 1, SeparableConv, [3, 1]]

- [19, 1, C3_DCNLite, [256, False]]

- [[26, 22, 25], 1, Detect, [nc, anchors]]

## References

[1] Kim J., Ko J., Choi H., Kim H. (2021). Printed Circuit Board Defect Detection Using Deep Learning via A Skip-Connected Convolutional Autoencoder. Sensors.

[2] Luo Q., Fang X., Su J., Zhou J., Zhou B., Yang C., Liu L., Gui W., Tian L. (2020). Automated Visual Defect Classification for Flat Steel Surface: A Survey. IEEE Trans. Instrum. Meas..

[3] Zheng X., Zheng S., Kong Y., Chen J. (2021). Recent advances in surface defect inspection of industrial products using deep learning techniques. Int. J. Adv. Manuf. Technol..

[4] Ling Q., Isa N.A.M. (2023). Printed Circuit Board Defect Detection Methods Based on Image Processing, Machine Learning and Deep Learning: A Survey. IEEE Access.

[5] Russakovsky O., Deng J., Su H., Krause J., Satheesh S., Ma S., Huang Z., Karpathy A., Khosla A., Bernstein M. (2015). ImageNet Large Scale Visual Recognition Challenge. Int. J. Comput. Vis..

[6] Zou Z., Chen K., Shi Z., Guo Y., Ye J. (2023). Object Detection in 20 Years: A Survey. Proc. IEEE.

[7] Huang G., Huang Y., Li H., Guan Z., Li X., Zhang G., Li W., Zheng X. (2024). An Improved YOLOv9 and Its Applications for Detecting Flexible Circuit Boards Connectors. Int. J. Comput. Intell. Syst..

[8] Adibhatla V.A., Chih H.-C., Hsu C.-C., Cheng J., Abbod M.F., Shieh J.-S. (2020). Defect Detection in Printed Circuit Boards Using You-Only-Look-Once Convolutional Neural Networks. Electronics.

[9] Chen H., Chen Z., Yu H. (2023). Enhanced YOLOv5: An Efficient Road Object Detection Method. Sensors.

[10] Adam M.A.A., Tapamo J.R. (2025). Enhancing YOLOv5 for Autonomous Driving: Efficient Attention-Based Object Detection on Edge Devices. J. Imaging.

[11] Shin Y., Shin H., Ok J., Back M., Youn J., Kim S. (2024). DCEF2-YOLO: Aerial Detection YOLO with Deformable Convolution–Efficient Feature Fusion for Small Target Detection. Remote Sens..

[12] Ryu J., Kwak D., Choi S. (2025). YOLOv8 with Post-Processing for Small Object Detection Enhancement. Appl. Sci..

[13] Zhu J., Chen J., He H., Bai W., Zhou T. (2025). CBACA-YOLOv5: A Symmetric and Asymmetric Attention-Driven Detection Framework for Citrus Leaf Disease Identification. Symmetry.

[14] Li Y., Miao N., Ma L., Shuang F., Huang X. (2023). Transformer for object detection: Review and benchmark. Eng. Appl. Artif. Intell..

[15] Arkin E., Yadikar N., Xu X., Aysa A., Ubul K. (2023). A survey: Object detection methods from CNN to transformer. Multimed. Tools Appl..

[16] Carion N., Massa F., Synnaeve G., Usunier N., Kirillov A., Zagoruyko S. End-to-End Object Detection with Transformers. Proceedings of the Computer Vision—ECCV 2020.

[17] Liu Z., Lin Y., Cao Y., Hu H., Wei Y., Zhang Z., Lin S., Guo B. Swin Transformer: Hierarchical Vision Transformer using Shifted Windows. Proceedings of the 2021 IEEE/CVF International Conference on Computer Vision (ICCV).

[18] Ancha V.K., Gonuguntla V., Vaddi R. (2025). TRSBi-YOLO: Transformer based lightweight and high-performance model for PCB defects detection. J. Supercomput..

[19] Chen W., Huang Z., Mu Q., Sun Y. (2022). PCB Defect Detection Method Based on Transformer-YOLO. IEEE Access.

[20] Luo T., Zhou Y., Shi D., Yun Q., Wang S., Zhang J., Ding G. (2025). A lightweight defect detection transformer for printed circuit boards combining image feature augmentation and refined cross-scale feature fusion. Eng. Appl. Artif. Intell..

[21] Wang Y., Wu B., Zhang L., Wang Z., Liu J., Dong J., Shi J. (2025). Enhanced PCB defect detection via HSA-RTDETR on RT-DETR. Sci. Rep..

[22] Peng J., Fan W., Lan S., Wang D. (2024). MDD-DETR: Lightweight Detection Algorithm for Printed Circuit Board Minor Defects. Electronics.

[23] Kang Z., Liao Y., Du S., Li H., Li Z. (2024). SE-CBAM-YOLOv7: An Improved Lightweight Attention Mechanism-Based YOLOv7 for Real-Time Detection of Small Aircraft Targets in Microsatellite Remote Sensing Imaging. Aerospace.

[24] Xu H., Wang L., Chen F. (2024). Advancements in Electric Vehicle PCB Inspection: Application of Multi-Scale CBAM, Partial Convolution, and NWD Loss in YOLOv5. World Electr. Veh. J..

[25] Kim M., Jeong J., Kim S. (2021). ECAP-YOLO: Efficient Channel Attention Pyramid YOLO for Small Object Detection in Aerial Image. Remote Sens..

[26] Shi P., Zhang Y., Cao Y., Sun J., Chen D., Kuang L. (2025). DVCW-YOLO for Printed Circuit Board Surface Defect Detection. Appl. Sci..

[27] Ni J., Zhu S., Tang G., Ke C., Wang T. (2024). A Small-Object Detection Model Based on Improved YOLOv8s for UAV Image Scenarios. Remote Sens..

[28] Xie M., Tang Q., Tian Y., Feng X., Shi H., Hao W. (2025). DCN-YOLO: A Small-Object Detection Paradigm for Remote Sensing Imagery Leveraging Dilated Convolutional Networks. Sensors.

[29] Xudong S., Yucheng W., Changxian L., Lifang S. (2024). WDC-YOLO: An improved YOLO model for small objects oriented printed circuit board defect detection. J. Electron. Imaging.

[30] Feng F., Hu Y., Li W., Yang F. (2024). Improved YOLOv8 algorithms for small object detection in aerial imagery. J. King Saud Univ.-Comput. Inf. Sci..

[31] Zhang D., Xu C., Chen J., Wang L., Deng B. (2025). YOLO-DC: Integrating deformable convolution and contextual fusion for high-performance object detection. Signal Process. Image Commun..

[32] Doherty J., Gardiner B., Kerr E., Siddique N. (2025). BiFPN-YOLO: One-stage object detection integrating Bi-Directional Feature Pyramid Networks. Pattern Recognit..

[33] Li N., Ye T., Zhou Z., Gao C., Zhang P. (2024). Enhanced YOLOv8 with BiFPN-SimAM for Precise Defect Detection in Miniature Capacitors. Appl. Sci..

[34] Yu S., Pan F., Zhang X., Zhou L., Zhang L., Wang J. (2025). A lightweight detection algorithm of PCB surface defects based on YOLO. PLoS ONE.

[35] Gao J., Geng X., Zhang Y., Wang R., Shao K. (2024). Augmented weighted bidirectional feature pyramid network for marine object detection. Expert Syst. Appl..

[36] Xie Y., Zhao Y. (2024). Lightweight improved YOLOv5 algorithm for PCB defect detection. J. Supercomput..

[37] Wang W., Xu J., Zhang R. (2025). Optimized small object detection in low resolution infrared images using super resolution and attention based feature fusion. PLoS ONE.

[38] Zhang J., Chen Z., Yan G., Wang Y., Hu B. (2023). Faster and Lightweight: An Improved YOLOv5 Object Detector for Remote Sensing Images. Remote Sens..

[39] Wang J., Chen K., Xu R., Liu Z., Loy C.C., Lin D. (2022). CARAFE++: Unified Content-Aware ReAssembly of FEatures. IEEE Trans. Pattern Anal. Mach. Intell..

[40] Zhao K., Xie B., Miao X., Xia J. (2023). LPO-YOLOv5s: A Lightweight Pouring Robot Object Detection Algorithm. Sensors.

[41] Sun M., Wang L., Jiang W., Dharejo F.A., Mao G., Timofte R. (2025). SF-YOLO: A Novel YOLO Framework for Small Object Detection in Aerial Scenes. IET Image Process..

[42] Liu X., Zhou S., Ma J., Sun Y., Zhang J., Zuo H. (2025). DFAS-YOLO: Dual Feature-Aware Sampling for Small-Object Detection in Remote Sensing Images. Remote Sens..

[43] Du Y., Jiang X. (2024). A Real-Time Small Target Vehicle Detection Algorithm with an Improved YOLOv5m Network Model. Comput. Mater. Contin..

[44] Liu N., Xu X., Gao Y., Zhao Y., Li H.-C. (2024). Semi-supervised object detection with uncurated unlabeled data for remote sensing images. Int. J. Appl. Earth Obs. Geoinf..

[45] Zhao T., Zeng Y., Fang Q., Xu X., Xie H. (2025). Semi-Supervised Object Detection for Remote Sensing Images Using Consistent Dense Pseudo-Labels. Remote Sens..

[46] Fu R., Chen C., Yan S., Wang X., Chen H. (2024). Consistency-based semi-supervised learning for oriented object detection. Know.-Based Syst..

[47] Wang M., Xu X., Liu H. (2025). A Semi-Supervised Object Detector Based on Adaptive Weighted Active Learning and Orthogonal Data Augmentation. Sensors.

[48] Zhang R., Yao M., Qiu Z., Zhang L., Li W., Shen Y. (2024). Wheat Teacher: A One-Stage Anchor-Based Semi-Supervised Wheat Head Detector Utilizing Pseudo-Labeling and Consistency Regularization Methods. Agriculture.

[49] Zhang R., Xu C., Xu F., Yang W., He G., Yu H., Xia G.-S. (2025). S3OD: Size-unbiased semi-supervised object detection in aerial images. ISPRS J. Photogramm. Remote Sens..

[50] Huang W., Wei P., Zhang M., Liu H. (2020). HRIPCB: A challenging dataset for PCB defects detection and classification. J. Eng..

[51] Ultralytics YOLOv8 Documentation and Model Overview. https://docs.ultralytics.com/models/yolov8/.

[52] Wang C.-Y., Yeh I.H., Mark Liao H.-Y. YOLOv9: Learning What You Want to Learn Using Programmable Gradient Information. Proceedings of the Computer Vision—ECCV 2024.

[53] Wang A., Chen H., Liu L., Chen K., Lin Z., Han J., Ding G. (2024). YOLOv10: Real-Time End-to-End Object Detection. Adv. Neural Inf. Process. Syst..

[54] Ultralytics YOLO11 Documentation and Models. https://docs.ultralytics.com/zh/models/yolo11.

